# 2129. Antimicrobial Activity of Cefepime in Combination with Taniborbactam Against Resistant Clinical Isolates from the United States 2018-2021

**DOI:** 10.1093/ofid/ofad500.1752

**Published:** 2023-11-27

**Authors:** Meredith Hackel, Mark G Wise, Daniel F Sahm

**Affiliations:** IHMA, Schaumburg, Illinois; IHMA, Schaumburg, Illinois; IHMA, Schaumburg, Illinois

## Abstract

**Background:**

Taniborbactam is a novel, investigational broad-spectrum β-lactamase inhibitor with direct inhibitory activity against both serine- and metallo-β-lactamases. Taniborbactam restores the activity of cefepime (FEP) against many difficult to treat organisms, including cephalosporin- and carbapenem-resistant Enterobacterales (EB) and *Pseudomonas aeruginosa* (PA). The activity of cefepime-taniborbactam (FTB) and comparator agents was evaluated against nonsusceptible (NS)/resistant (R) clinical isolates of EB and PA from the United States (US) collected in global surveillance.

**Methods:**

MICs of FTB (taniborbactam fixed at 4 µg/mL) and comparators were determined using the CLSI reference method against EB (n=4,220) and PA (n=1,222) from the US collected in 2018-2021. NS/R phenotypes were based on 2023 CLSI breakpoints (EUCAST breakpoint for meropenem-vaborbactam [MEV] against PA). Multidrug R (MDR) was defined as R to ≥1 agent from ≥3 drug classes.

**Results:**

Overall, 13.1% and 10.3% of EB isolates were NS to FEP and piperacillin-tazobactam (TZP), respectively (Table). FTB had potent activity against all EB (MIC_50/90_, 0.03/0.12 µg/mL; 99.9% inhibited at ≤16 µg/mL). FTB maintained activity against NS/R subsets of EB (MIC_90_ range, 1 to 8 µg/mL; 90.0% to 99.3% inhibited at ≤16 µg/mL) including MDR isolates (MIC_90_, 2 µg/mL; 98.4% inhibited at ≤16 µg/mL). FTB was the most active tested agent against PA overall (MIC_50/90_, 2/8 µg/mL; 97.7% inhibited at ≤16 µg/mL). Among meropenem (MEM)-NS isolates, 91.2% were inhibited by FTB at ≤16 µg/mL compared to 76.6% susceptible to ceftolozane-tazobactam (CT). FTB at ≤16 µg/mL inhibited 74.2% of CT NS isolates of PA whereas 47.2% of isolates of this phenotype were susceptible to ceftazidime-avibactam (CZA). Against MDR PA (13.4% of all PA), FTB (MIC_50/90_, 8/32 µg/mL) inhibited 85.4% of isolates at ≤16 µg/mL compared to 59.1% that were susceptible to CT.

**Figure:**
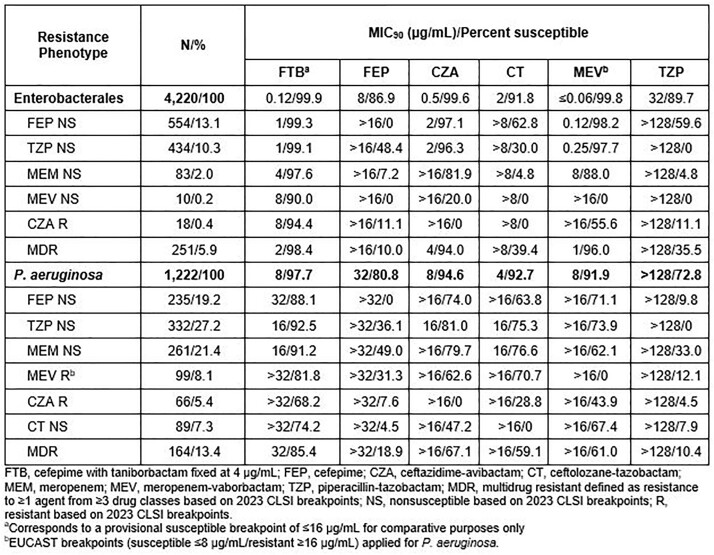

**Conclusion:**

Cefepime-taniborbactam had potent *in vitro* activity against recent EB and PA from the US, including MDR isolates and isolates NS to FEP, MEM, TZP, CZA, CT, and/or MEV. These data support continued development of cefepime-taniborbactam as a potential treatment option for challenging infections due to resistant Gram-negative pathogens.

**Disclosures:**

**Meredith Hackel, PhD**, Pfizer Inc.: Honoraria|Venatorx: Paid fees for conducting the study and abstract preparation **Mark G Wise, PhD**, Merck & Co., Inc.: Honoraria|Pfizer Inc.: Honoraria|Venatorx: Paid fees for conducting the study and abstract preparation **Daniel F. Sahm, PhD**, Merck & Co., Inc.: Honoraria|Pfizer Inc.: Honoraria|Venatorx: Paid fees for conducting the study and abstract preparation

